# Evaluating the effectiveness of the Ministry of Health restriction policy on seasonal antibiotic consumption trends in Saudi Arabia, 2016–2020

**DOI:** 10.3389/fphar.2023.1242087

**Published:** 2023-11-30

**Authors:** Khaloud O. Alzahrani, Saeed M. Alshahrani, Sulaiman M. Alajel

**Affiliations:** ^1^ Molecular Biology Division, Reference Laboratory for Microbiology, Executive Department of Reference Laboratories, Research and Laboratories Sector, Saudi Food and Drug Authority (SFDA), Riyadh, Saudi Arabia; ^2^ Department of Public Health, College of Applied Medical Sciences, King Khalid University, Abha, Saudi Arabia; ^3^ Reference Laboratory for Microbiology, Executive Department of Reference Laboratories, Research and Laboratories Sector, Saudi Food and Drug Authority (SFDA), Riyadh, Saudi Arabia

**Keywords:** antibiotic consumption, seasonal variation, IQVIA-MIDAS, defined daily dose DDD/1,000 inhabitants per day, anatomical therapeutic chemical classification system (ATC)

## Abstract

**Background:** Understanding antibiotic consumption patterns over time is essential to optimize prescribing practices and minimizing antimicrobial resistance. This study aimed to determine whether the antibiotics restriction policy launched by the Saudi Ministry of Health in April 2018 has impacted antibiotic use by assessing changes and seasonal variations following policy enforcement.

**Methods:** Quarterly sales data of J01 antibacterial for systemic use in standard units were obtained from the IQVIA-MIDAS database, spanning from the first quarter of 2016 to the last quarter of 2020. Antibiotics consumption was measured in defined daily doses per 1,000 inhabitant per day- in a quarter (DDDdq). A comparative analysis of antibiotic consumption pre- and post-policy periods introduction was conducted by computing the average consumption values for each period. Statistical comparison of the mean differences between the two periods were then made using independent samples t-test, Mann-Whitney U Test where needed. Time series analysis was employed to estimate the projected antibiotic consumption in the post-policy period if the restriction policy had not been implemented, which was then compared to actual consumption values to evaluate the effectiveness of the restriction policy.

**Results:** During the pre-policy, there were seasonal trends of the total and oral antibiotic consumption through quarters, with higher consumption observed in the first and fourth quarters. In contrast, parenteral antibiotic consumption did not appear to follow a clear seasonal pattern. Following the restriction policy, there was a significant reduction in total and oral antibiotic use, with mean reductions of −96.9 DDDdq (*p*-value = 0.002) and −98 DDDdq (*p*-value = 0.002), respectively. Conversely, a significant increase in parenteral antibiotic consumption was observed with a mean increase of +1.4 DDDdq (*p*-value < 0.0001). The comparison between the forecasted and actual models showed that the actual antibiotics consumption for total, oral, and parenteral were lower than the corresponding forecasted values by 30%, 31%, and 34%, respectively.

**Conclusion:** Overall, our analysis of antibiotics consumption from 2016 to 2020 displays great success for the policy implemented by the Saudi Ministry of Health in significantly reducing the total and oral use of antibiotics. However, future studies are needed to explore the increased consumption of the parenteral antibiotics as well as the persistent high consumption patterns during the fall and winter months even after the implementation of the restriction policy.

## Introduction

The current global increase in antimicrobial resistance (AMR) coupled with the shortage in developing new antibiotics poses serious public health and economic challenges (Ventola, 2015). Projections indicate that AMR could become the leading cause of death globally by 2050, unless it is adequately addressed (de Kraker et al., 2016). Several investigations conducted in Saudi Arabia highlighted the significant burden of AMR and its growing prevalence among Gram-negative and Gram-positive bacteria (Al Johani et al., 2010; Aly and Balkhy, 2012; Zowawi et al., 2013; Yezli et al., 2014; Balkhy et al., 2020; Al Mutair et al., 2021). Increased levels of AMR could be arise from various factors, such as inadequate therapy duration, inappropriate dosages, and irrational fixed-dose drug combinations, but the most significant factor is the misuse of antibiotics (WHO, 2018).

During previous years, the prohibition of over-the-counter antibiotics sales in private pharmacies was not enforced in Saudi Arabia, and non-prescribed use of antibiotics was common practice for unnecessary self-limiting conditions such as common cold (Nafisah et al., 2017). A study conducted by AlKhamees et al. (2018) showed that amoxicillin/clavulanic acid belonging to the J01C group, was the second most used medication in Saudi Arabia after painkillers. Several studies from various regions in Saudi Arabia have indicated a lack of public knowledge and widespread misuse of antibiotics (Al-Rukban and Khalil, 2012; Alharbi et al., 2017; Awadh et al., 2017; Nafisah et al., 2017; Alhomoud et al., 2018; Ballal, 2019; Yagoub et al., 2019; Zaidi et al., 2021). Similarly, inadequate knowledge and poor practices among healthcare professionals have been reported (Alzahrani et al., 2020; Baraka et al., 2021). In 2016, it was reported that the pharmaceutical expenditure in Saudi Arabia reached 7 billion US dollars, with nearly 2 billion US dollars spent on antibiotics (Almeleebia et al., 2021).

Global stewardship programs were implemented in many countries to reduce overall antibiotic prescribing and reduce the burden of AMR resistance. In April 2018, the Saudi Ministry of Health (MOH) initiated a nationwide antibiotics restriction policy, in which pharmacies are strictly prohibited from dispensing antibiotics without a prescription. Failure to adhere to the regulations could lead to severe penalties, including license revocation, fines up to 100 thousand riyals, and imprisonment for up to 6 months (MOH, 2018). Despite the large volume of antibiotic consumption, only little is known about usage patterns. Detailed surveillance of antibiotic use in the community is one of the strategies that could be used to guide and control antibiotic overuse and misuse.

Unfortunately, the effectiveness of governmental interventions to enhance antibiotic use in Saudi Arabia has not been thoroughly investigated in Saudi Arabia. One recent study conducted by Al-Jedai et al. (2022), conducted from May 2017 to May 2019, showed that MOH policy enforcement has only an immediate and temporary effect on reducing antimicrobial use in community pharmacies across Saudi Arabia. Given the study’s limited timeframe—covering 1 year before and 1 year after MOH policy implementation—the assessment of the policy’s effectiveness and sustainability may be questioned. Moreover, the analyses used in this study have not fully addressed the potential seasonality effect of antibiotic consumption.

Therefore, this study aims to assess the impact of the Saudi MOH restriction policy over a more extended period (5 years period; 2016–2020). It also seeks to describe trends and seasonal variations in antibiotic use to determine opportunities for public health intervention where seasonal peaks may represent increases in inappropriate use. The extended data timeframe period will also provide a more accurate assessment of the policy’s long-term effectiveness and sustainability. We finally attempted to assess the potential effect of the COVID-19 pandemic on antibiotic use patterns during the last three-quarters of 2020. The finding from this study can be utilized by policymakers to continue monitoring antibiotic misuse, developing rational-use policies, and establishing a baseline for future assessment.

## Materials and methods

### Study source and setting

This quasi-experimental study utilizes 5 years retrospective data on antibiotic consumption obtained from the IQVIA-MIDAS database from 2016 to 2020. Data were extracted quarterly, with Q3 2018 marking the MOH intervention’s onset. The IQVIA database provides estimates of antibiotic use based on the volume sold in retail and hospital pharmacies through national surveys conducted along pharmaceutical sales distribution channels (from manufacturer to wholesaler to retailer). All antimicrobial drugs belonging to antibacterials for systemic use (J01) were included in this study regardless of brand or generic name or route of administration: oral and parenteral (intravenous/intramuscular). However, topical, ophthalmic, otic, and local (intravaginal and pessaries) antimicrobial data were excluded. These antibiotics constituted 62 different antibiotic molecule types, grouped into eight classes belonging to J01 group according to the Anatomical Therapeutic Chemical (ATC-3) classification system (World Health Organization, 2023) (https://www.whocc.no/atc/structure_and_principles/). This group (J01) is further divided into eight pharmacological subgroups: tetracyclines (J01A), beta-lactam and penicillins (J01C), cephalosporins (J01D), sulfonamides and trimethoprim (J01E), macrolides (J01F), aminoglycosides (J01G), quinolones (J01M), and urinary antiseptics (J01X). Information for amphenicols (J01B) and combinations of antibacterials (J01R) are unavailable. The distribution of antibiotics into classes is listed in Table 1.

**TABLE 1:** J01 systemic antibacterials list.

ATC3- antibiotics classification	Molecule type list
J01A Tetracyclines	Doxycycline, Tetracycline, Minocycline
J01C Beta-lactam antibacterials, penicillins	Amoxicillin, Amoxicillin-Clavulanic Acid, Ampicillin, Ampicillin-Sulbactam, Piperacillin-Tazobactam, Flucloxacillin, Cloxacillin, Phenoxymethylpenicillin, Benzylpenicillin
J01D Other Beta-lactam antibacterials	Avibactam-Ceftazidime, Cefaclor, Cefadroxil, Cefalexin, Cefazolin, Cefdinir, Cefditoren Pivoxil, Cefepime, Cefixime, Cefoperazone, Cefotaxime, Cefoxitin, Cefpodoxime Proxetil, Ceftaroline Fosamil, Cefprozil, Cefprozil, Cefradine, Ceftazidime, Ceftobiprole Medocaril, Ceftolozane-Tazobactam, Ceftriaxone, Cefuroxime Axetil, Aztreonam, Cilastatin-Imipenem, Ertapenem, Meropenem
J01E Sulfonamides and Trimethoprim	Sulfamethoxazole-Trimethoprim, Trimethoprim
J01F Macrolides, Lincosamides and Streptogramins	Azithromycin, Clarithromycin, Clindamycin, Erythromycin, Josamycin
J01G Aminoglycoside antibacterial	Amikacin, Gentamicin, Tobramycin
J01M Quinolone antibacterials	Ciprofloxacin, Gemifloxacin, Levofloxacin, Lomefloxacin, Moxifloxacin, Ofloxacin
J01X Other antibacterials	Linezolid, Daptomycin, Colistin, Tedizolid, Teicoplanin, Tigecycline, Vancomycin

### Measures of antibiotic consumption rate

Antibiotic consumption was estimated based on sales volume reported in standard units (SUs). IQVIA-MIDAS defines one SU as a single tablet, capsule, ampoule, vial, or 5 mL liquid preparation for oral consumption. Quarterly data on antibiotic use were measured in defined daily doses DDD per 1,000 inhabitant per day in quarter (DDDdq) from the first quarter of 2016 to the last quarter of 2020 using the ATC-3 Classification System (ATC/DDD, 2022) developed by the WHO Collaborating Centre for Drug Statistics Methodology (WHO, 2009). The annual antibiotic consumption rate in Saudi Arabia, expressed in DDDdq, was calculated using population estimates from the World Bank (2016–2020).

$$DDD/1000\;inhabitant\;per\;day=\frac{Streangth\;X\;Quantity\;X\;Units\;sold\;X\;1000}{DDD\;\left(mg\right)\;X\;Population\;X\;Number\;of\;days}$$

Winter months were defined as the first (Q1: January, February, and March) and fourth (Q4: October, November, and December) quarters of the tested years. Summer months were defined as the second (Q2: April, May, and June) and third (Q3: July, August, and September) quarters of the tested years.

### MOH restriction policy (the intervention)

Prior to April 2018, antibiotics were accessible over the counter in Saudi Arabia, despite Ministry of Health (MOH) advisories against such practices, with limited pharmacy adherence. To address this, the MOH launched a nationwide restriction policy in April 2018 prohibiting pharmacies from dispensing antibiotics without a prescription. This policy carried substantial penalties for non-compliance, including potential license revocation, fines up to 100,000 Saudi Riyals (approximately $26,700 USD), and imprisonment for up to 6 months. Complementing this policy, the MOH also launched extensive campaigns to educate both the public and healthcare providers about rationalizing antibiotics use, utilizing various platforms including malls, healthcare institutions, and social media (MOH, 2018).

### Data analysis

Descriptive statistics were used to evaluate antibiotic consumption in the form of the DDDdq across the defined pre- and post-policy periods to capture the seasonal variations and assess the effectiveness of the restriction policy. We refer to the period before the restriction policy as “*Pre-Policy*” and the period after the restriction policy as “*Post-Policy.*” The variables of interest in this analysis include the total, oral, and parenteral antibiotic consumption, alongside consumption for each antibiotic class as shown in Table 1. To compare the consumption between pre-policy and post-policy periods, we computed the average consumption within each period; then, we used independent samples t-test, Mann Whitney U Test where needed, to compare the mean difference between the two periods. We also calculated the percentage of change in consumption from pre-policy to post-policy periods. Furthermore, we assessed the effectiveness of the restriction policy using time series analysis to forecast antibiotic consumption in the post-policy period if the restriction policy had not been implemented. The forecasted consumption was then compared with the actual consumption during the corresponding period to assess potential differences. Stationary R-squared was used to assessed the seasonal variation and the forecast model reliability. The model was proved reliable and able to detect seasonality for total and oral antibiotic consumption but not for parenteral antibiotic consumption. The potential effect of the COVID-19 pandemic on antibiotic consumption during the post-policy period was evaluated by comparing antibiotic consumption from Q3 2018 to Q1 2020 with the remaining quarters of 2020 (Q2–Q4, 2020). Analysis was conducted using IBM SPSS version 21.0 (SPSS Inc., Chicago, IL, United States), with a significance threshold of *p* < 0.05.

## Results

**FIGURE 1:**
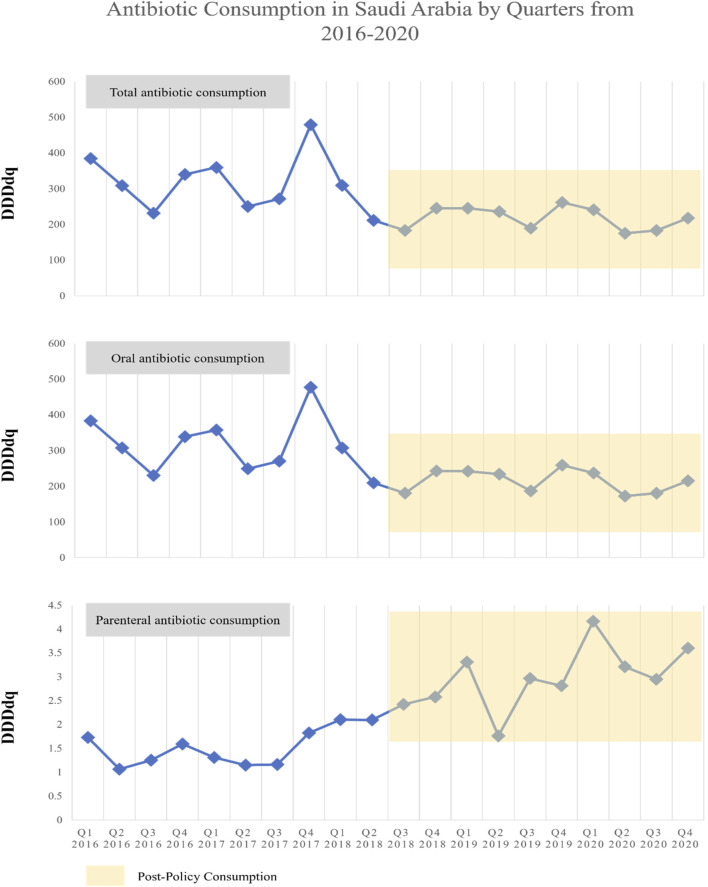
The antibiotic consumption in Saudi Arabia by quarters from 2016 to 2020.

The average DDDdq of the total, oral, and parenteral antibiotic consumptions in Saudi Arabia during the pre-policy period were 314.5, 313.3, and 1.5, respectively, as compared to the correspondent values during the post-policy (217.6, 215.3, and 2.9, respectively). Pre-policy data indicated seasonal consumption trends for total and oral antibiotics through quarters, with peaks observed in Q1 and Q4. In contrast, parenteral antibiotic consumption did not exhibit a clear seasonal pattern. During the post-policy period, noticeable declines in total and oral antibiotic consumption were observed, while an increase in parenteral antibiotic consumption was noted (Figure 1).

**FIGURE 2:**
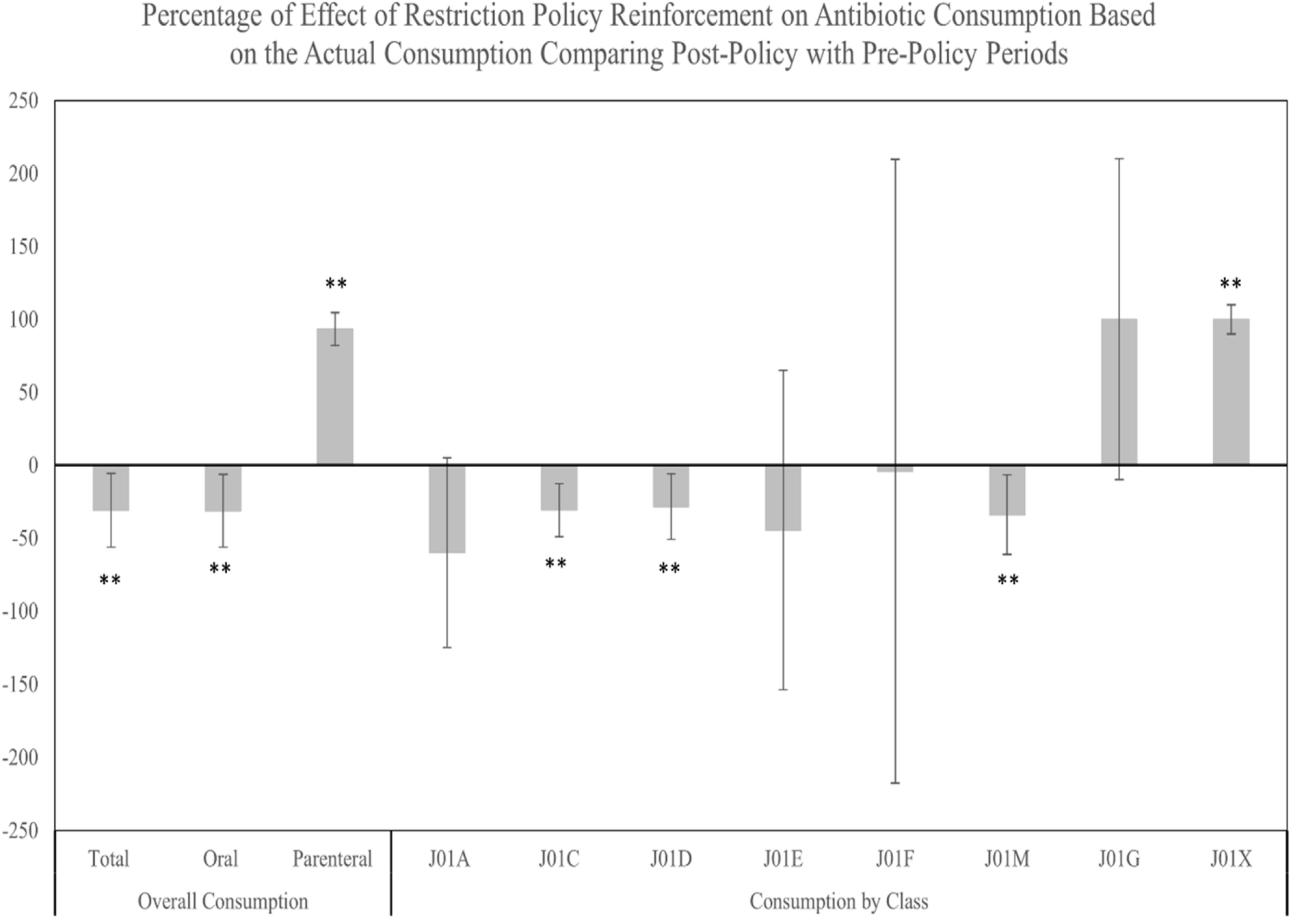
Percentage of change in the antibiotic actual consumption from the pre-policy period to the post-policy period. ** Significant at *α* < 0.05. Error bars were derived from relative standard deviation (RSD).

Results from mean comparisons between pre- and post-policy periods indicated that the total and oral antibiotic consumptions were significantly decreased after the restriction policy with mean changes of −96.9 DDDdq (*p*-value = 0.002) and −98 DDDdq (*p*-value = 0.002), respectively. Conversely, a significant increase after the restriction policy in the parenteral antibiotic consumption was observed with a mean change of +1.4 DDDdq (*p*-value < 0.0001) (Supplementary Table S1). In addition, the percentage change in actual antibiotic consumption reflected substantial decreases in total and oral consumptions post-policy, while parenteral consumption notably increased (Figure 2).

**FIGURE 3:**
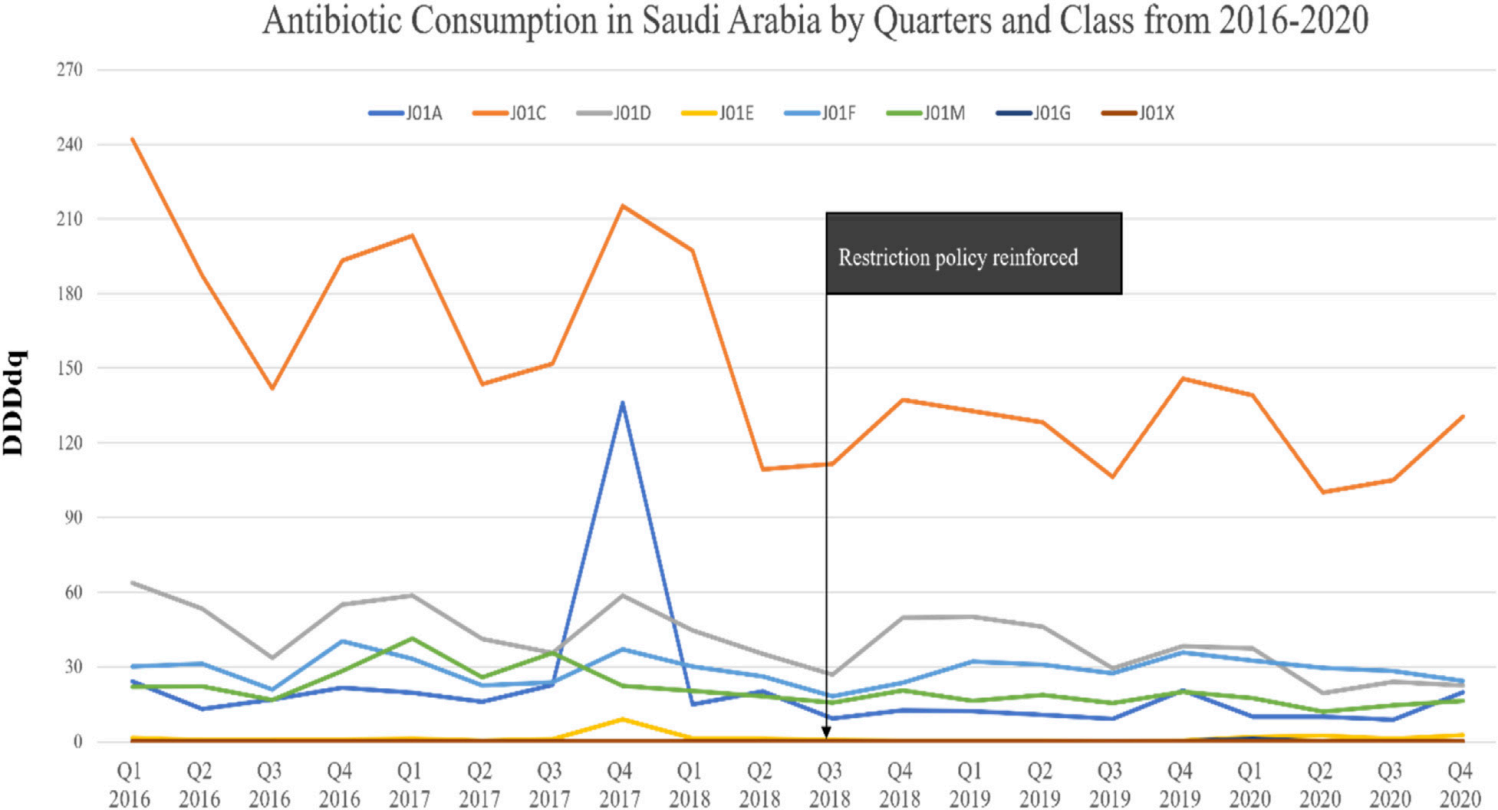
The antibiotic consumption in Saudi Arabia by quarters and class from 2016 to 2020.

Analyzing consumption by antibiotic class, similar seasonal trends of total and oral antibiotic consumption were observed during pre- and post-policy periods, with J01C exhibiting the highest DDDdq among all classes. In the post-policy period, significant decreases were observed predominantly in J01C, followed by J01D, and J01M classes, with mean changes of −54.8 DDDdq (*p* = .002), −13.6 DDDdq (*p* = 0.016), and −8.6 DDDdq (*p* = 0.007), respectively. The J01X class, however, showed a significant uptick, with a mean change of +0.1 DDDdq (*p* = 0.003) (Supplementary Table S1). The percentage of change in the antibiotic actual consumption from the pre-policy period to the post-policy period demonstrated significant decreases in the consumption of J01C, J01D, and J01M classes. In contrast, a significant increase in the percentage of change was observed for the consumption of the J01X class. No significant changes in the percentage of consumption were observed for the J01A, J01E, J01F, and J01G classes (Figure 3).

**FIGURE 4:**
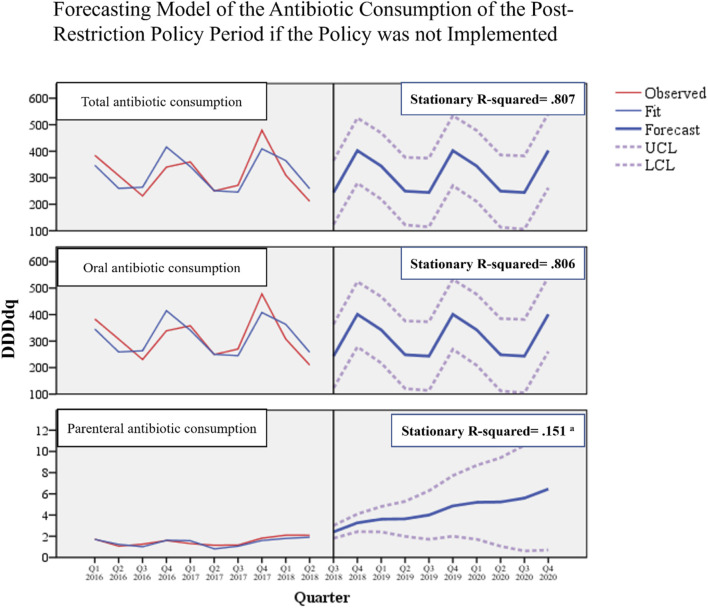
Forecasting model of antibiotic consumption during the post-restriction policy period if the policy was not implemented. ^a^ Poor stationary R-squared indicates unreliability of the forecasting model due to the absence of seasonality.

Regarding the time series forecasting model we used to assess the seasonal variations of antibiotic consumption, the model was considerably reliable in detecting the seasonality trends of the post-policy consumption for total (stationary R-squared = 0.807) and oral (stationary R-squared = 0.806) antibiotics if the policy were not implemented. The model was, however, unreliable in detecting the seasonality for parenteral antibiotic consumption due to poor stationary R-squared, which indicates an absence of seasonality (Figure 4).

**FIGURE 5:**
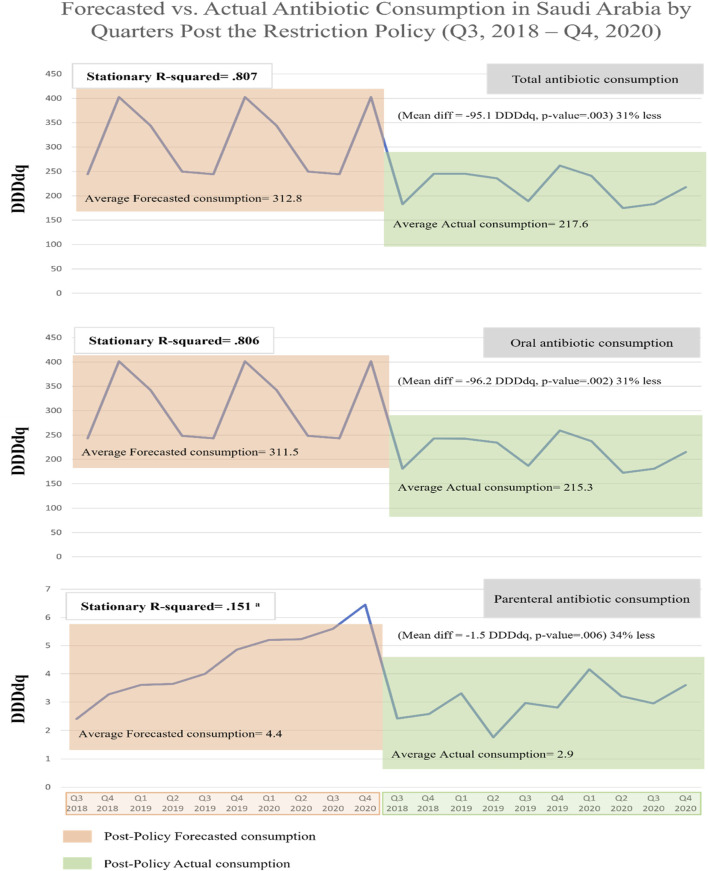
Forecasted vs. actual antibiotic consumption in Saudi Arabia by Quarters during the post-restriction policy (Q3, 2018–Q4, 2020). ^a^ Poor stationary R-squared indicates unreliability of the forecasting model due to the absence of seasonality.

When comparing forecasted versus actual antibiotic consumption post-restriction, we observed that actual consumptions were lower than corresponding forecasted for total consumption by 30% (mean difference −95.1 DDDdq, *p* = 0.003), for oral consumption by 31% (mean difference −96.2 DDDdq, *p* = 0.002), and for parenteral consumption by 34% (mean difference −1.5 DDDdq, *p* = 0.006) (Figure 5). These results suggest that the restriction policy markedly reduced total and oral antibiotic consumption, which would have otherwise continued to rise.

**FIGURE 6:**
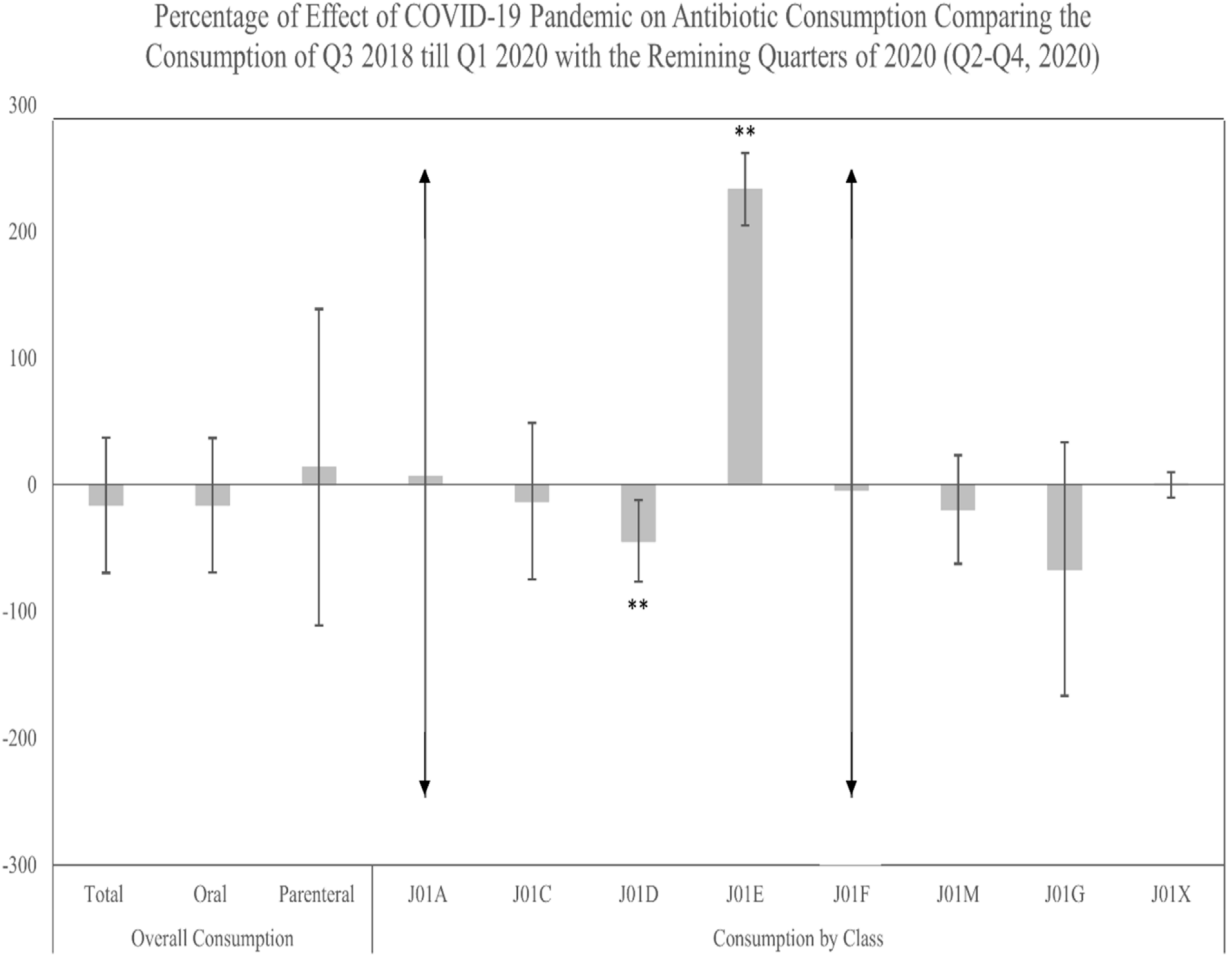
Percentage of change in the antibiotic actual consumption after the implementation of the restriction policy comparing the pre-COVID-19 period (Q3, 2018–Q1, 2020) to the post-COVID-19 period (Q2–Q4, 2020). ** Significant at *α* < 0.05. Error bars were derived from relative standard deviation (RSD).

To assess the COVID-19 pandemic’s potential effect on antibiotic consumption during the post-policy period, we analyzed data from Q3 2018 to Q1 2020 against the latter quarters of 2020 (Q2–Q4) (Supplementary Table S2). The findings indicated no significant pandemic-related impacts on total, oral, or parenteral antibiotic consumption. Similarly, no significant impacts were observed for the consumption of J01A, J01C, J01F, J01M, J01G, and J01X classes. However, a significant decrease in J01D class consumption and an increase in J01E class consumption during the pandemic period were noted (Figure 6).

## Discussion

This study aimed to measure the changes in antibiotic consumption—considering the seasonal variations of consumption—before and after the implementation of the MOH restriction policy in Saudi Arabia in mid- 2018. It also assessed the effect of the COVID-19 pandemic on antibiotic consumption during the post-policy period. We noted a substantial decrease in the consumption of antibiotics for systemic use (ATC J01), from 314.5 DDDdq during the pre-policy phase to 217.6 DDDdq in the post-policy phase. Our forecasting analysis of antibiotic consumption also underscores the MOH policy’s success in significantly reducing the overall use of antibiotics after comparing the actual consumption values during the post-policy period with the forecasted consumption values if the policy had not been implemented. Seasonal fluctuations were observed over the study period—including both pre- and post-policy periods, where the highest dip in antibiotic use was in Q2 and Q3, and the highest peaks were during Q1 and Q4. The continued elevation in antibiotic consumption during Q1 and Q4 (Fall and winter months) in the post-policy period—although at a much lower rate than the pre-policy period—could be attributed to either physicians’ poor practice in attempting to treat upper respiratory viral infections with antibiotics or bacterial complications arising from viral infections that required antibiotics therapy. Another potential reason could be related to the so-called “Wasfaty,” an electronic prescription system implemented by the Saudi MOH in 2018 (Almaghaslah et al., 2022). This system enables physicians to prescribe medications remotely at any time by entering a patient’s ID number, often without an in-person consultation, after which patients can retrieve their prescriptions from any nearby pharmacy. Such convenience, while innovative, could inadvertently contribute to the misuse of antibiotics during the post-policy period. Hence, a thorough evaluation of “Wasfaty” application and its adherence to standard prescription protocols is essential to ensure it does not compromise antibiotic restriction post-policy implementation.

The reduction in use was mainly led by (ATC group J01C) with 30.7% less in the post-policy period compared to the pre-policy period, indicating that the inappropriate use of penicillin may have diminished after the restrictions were enforced. Penicillin is the most commonly used antibiotic in Saudi Arabia and many parts of the world such as European Union countries (Bruyndonckx et al., 2021), so the restriction policy would have more impact. Our finding is consistent with similar studies from other countries such as Brazil that found an approximately 30% reduction in penicillin consumption after a restriction policy was implemented (Lopes-Júnior et al., 2015).

A sudden increase in the (ATC group J01A; Tetracyclines) consumption was observed in the last quarter of 2017 during the pre-policy phase. Despite our thorough analysis, the specific factors contributing to this sudden increase remain indeterminate and challenging. It is important to acknowledge that in complex healthcare systems and pharmaceutical landscapes, not all variations in consumption patterns can be definitively explained. However, several non-exclusive factors such as epidemiological trends, policy changes, prescribing behaviors, availability, or even the marketing practices of pharmaceutical companies giving the fact that J01A is one of the cheapest drugs, all these reasons could influence these pattern sudden changes. Unfortunately, we are unable to distinguish between these factors since the IQVIA data did not include information on prescription indications or microbiological data. This highlights the necessity for ongoing research where further investigations, could provide additional insights into these consumption dynamics especially for J01A group.

Surprisingly, antibiotic consumption of ATC group J01X significantly increased after implementing the restriction policy. This is possibly because the J01X antibiotic class is categorized as a reserve group according to the AWaRe (access, watch, reserve) classification system (e,g., glycopeptides, polymyxins, Imidazole derivatives, etc.), which are often used as a last resort for treating serious infections caused by multidrug-resistant bacteria (Van Boeckel et al., 2014). Increased consumption of J0X antibiotics has been observed in many countries such as the USA, France, Germany, India, the Republic of Korea, China, the UK and was correlated with the emergence of methicillin-resistant *Staphylococcus aureus* (MRSA) and extended-spectrum beta-lactamase (ESBL) producing Gram-negative resistant bacteria (Truter et al., 1996; Van Boeckel et al., 2014; Yoon et al., 2015; Qu et al., 2018; Donkor and Codjoe, 2019). Similarly, we believe that the increase in J01X prescriptions, especially vancomycin and colistin in Saudi Arabia, may be related to an increase in similar cases (Zowawi, 2016). Other potential causes for the rise in J01X consumption in recent years include the emergence and spread of multidrug-resistant bacteria, poor prescribing habits, and the shortage of effective alternative treatments (Banawas et al., 2018; Cara et al., 2018). Future direction in Saudi Arabia should include long-term surveillance and extensive research to elucidate the reasons behind the increase in reserve antibiotic use and to develop targeted interventions.

Although the MOH restriction policy was solely intended to limit the use of over-the-counter antibiotics, a substantial increase in the use of parenteral antibiotics following the implementation of the policy was observed. The overuse of parenteral antibiotics in this study cannot be ignored and poses concerns. Several potential factors could explain the reported rise in the usage of parenteral antibiotics. One key reason is parenteral antibiotics use was very low (Mean of consumption = 4.4 DDDdq) prior to the implementation of the MOH restriction policy, so any increase in use would be proportionately large. Another reason is the tendency of patients to delay seeking medical attention for infections when accessibility to oral antibiotics is constrained. This may cause the infection to worsen to the point where parenteral antibiotics are a better alternative for treatment. Other patients may have difficulty adhering to oral antibiotic regimens, leading to treatment failure, the development of AMR, and the need for more potent parenteral antibiotics. Another factor contributing to the increase in parenteral antibiotic use after the implementation of a restriction policy is the inappropriate prescribing practice by healthcare providers. Healthcare workers may be more likely to prescribe parenteral antibiotics as a precautionary measure, especially if they do not have time to diagnose bacterial infections or are faced with uncertainty in difficult cases (Pulcini et al., 2019). Usually, the antibiotic prescription is a difficult procedure influenced by multiple factors such as physician attitudes, patient symptoms, and time constraints (Rodrigues et al., 2013). Another reason could be the lack of proper education and training programs among healthcare providers on antibiotic prescribing guidelines. Therefore, more in-depth studies are needed to explore indications of parenteral use to understand why these antibiotics are so popular among patients and physicians. It is crucial for healthcare providers and policymakers to carefully consider the potential impacts of restriction policies on antibiotic use and to implement strategies to mitigate any negative effects or unintended consequences.

In March 2020, with the arrival of the COVID-19 pandemic caused by SARS-CoV-2, multiple aspects of treating respiratory tract infections, including antibiotics, were affected by the global health crisis. Unfortunately, due to the lack of purchase unit data for the years 2021 and 2022, we could not comprehensively evaluate the pandemic’s possible impact on antibiotic use. However, we sought to use data from the final three-quarters of 2020 to investigate any potential influence of COVID-19 on antibiotic usage during these three-quarters compared to previous quarters since the restriction policy was implemented. Overall, we found no statistically significant changes in total, oral, or parenteral antibiotic usage. No notable changes in the consumption of the J01A, J01C, J01F, J01M, J01G, and J01X groups were detected, except for the J01D group, which continued to decrease throughout the pandemic. Our findings agree to previous research, which indicated that the global trend of antibiotic consumption remained steady over the study period (Guisado-Gil et al., 2020). These observations could be attributed to Saudi governmental population-forced measures (mask usage, social distance, hand sanitizer) to minimize respiratory illnesses, which correlate to restricted antibiotic use. Interestingly, however, we found that (ATC group J01E) consumption has significantly increased during the pandemic. The increased use of this group, which possess immunomodulatory and anti-inflammatory properties, could be due to their perceived benefits for treating severe COVID-19 cases, especially given the early recommendations against the use of non-steroidal anti-inflammatory drugs (NSAIDs) for COVID-19 patients (Micallef et al., 2020). One study has suggested the importance of using Trimethoprim therapy (ATC J01E) to reduce acute lung injury in patients with severe COVID-19, thereby reducing the need for ventilatory support and improving outcomes (Varney et al., 2023). However, it is important to acknowledge that this approach was part of an evolving therapeutic strategy in response to an emergent unknown and new viral pandemic, rather than a reflection of established clinical efficacy. In Saudi Arabia, the early stage of the COVID-19 pandemic presented unprecedented challenges to healthcare providers like the situation worldwide. There was significant uncertainty regarding effective treatment standard protocols for COVID-19, leading to varied therapeutic approaches based on emerging evidence and clinical trials. Nevertheless, future research is needed to assess patterns of antimicrobial use before, during, and after the COVID-19 pandemic in Saudi Arabia.

To the best of our knowledge, the current study is the first study by far that investigated the changes in antibiotic consumption—considering the seasonal variations of consumption—before and after the implementation of the MOH restriction policy in Saudi Arabia, yet several limitations need to be addressed. Firstly, the IQVIA database used in the study does not differentiate between sales of prescription and non-prescription antibiotics. This means that the sales data may not accurately reflect the real consumption status in Saudi Arabia, especially in the pre-policy phase. Secondly, the IQVIA database includes antibiotics purchasing units from both private and public healthcare institutions; meaning it does not segregate the antibiotic purchasing units by the private pharmacies from public ones, so the results cannot reflect the private sector’s contribution to total antibiotic consumption. Finally, the IQVIA antibiotics purchasing units do not provide information on consumption by specific groups, such as age or gender, which could have been useful for better planning antibiotics stewardship activities. In general, the IQVIA database gives information mainly on antibiotics used across the nation, where most of the antibiotic usage occurs. Future research must, therefore, take hospital data into account to obtain a fuller picture of antibiotic utilization in Saudi Arabia.

## Conclusion

We revealed a positive impact of the MOH restriction policy that aimed to reduce the misuse and overuse of antibiotics utilizing a sophisticated analysis approach such as seasonal variations. A more pronounced impact was observed on oral antibiotic consumption. Concerns remain regarding the increased consumption of parenteral antibiotics during the post-policy period. Future research should longitudinally assess the MOH policy’s sustainability and explore the underlying reasons for potential non-compliance.

## Data Availability

The data analyzed in this study is subject to the following licenses/restrictions: IQVIA-MIDAS data. Requests to access these datasets should be directed to smotibi@sfda.gov.sa.
