# Supplementary material for: Evaluating the effectiveness of the Ministry of Health restriction policy on seasonal antibiotic consumption trends in Saudi Arabia, 2016–2020
Source: Front Pharmacol. 2023 Nov 30;14:1242087. doi: 10.3389/fphar.2023.1242087 (PMC10720327; doi:10.3389/fphar.2023.1242087)
Supplement: Supplementary file 1 [file DataSheet1.PDF]

Supplementary materials of submitted manuscript entitled “Evaluating the Effectiveness of the Ministry of Health Restriction Policy on Seasonal Antibiotic Consumption Trends in Saudi Arabia, 2016-2020”

Table 1. Effect of Restriction Policy Reinforcement on Antibiotic Consumption Based on the Actual Consumption Comparing Post-Policy with Pre-Policy Periods

Consumption	Period	Mean (SD)		P-value *
Total	Post-Policy	217.6	(32.3)	
	Pre-Policy	314.5	(80.5)	
	Change	-96.9	(27.4) [a]	**0.002 [b]**
Oral	Post-Policy	215.3	(32.2)	
	Pre-Policy	313.3	(80.4)	
	Change	-98.0	(27.4) [a]	**0.002 [b]**
Parenteral	Post-Policy	2.9	(0.7)	
	Pre-Policy	1.5	(0.4)	
	Change	+1.4	(0.2) [a]	**< 0.0001 [b]**
**Consumption by Class**				
J01A	Post-Policy	12.3	(4.3)	
	Pre-Policy	30.6	(37.3)	
	Change	-18.3	(11.9) [a]	0.141
J01C	Post-Policy	123.7	(16.4)	
	Pre-Policy	178.5	(40.4)	
	Change	-54.8	(13.8) [a]	**0.002 [b]**
J01D	Post-Policy	34.4	(11.5)	
	Pre-Policy	48.0	(11.2)	
	Change	-13.6	(5.1) [a]	0.**016 [b]**
J01E	Post-Policy	1.0	(0.9)	
	Pre-Policy	1.8	(2.6)	
	Change	-0.8	(0.9) [a]	0.384
J01F	Post-Policy	28.3	(5.1)	
	Pre-Policy	29.6	(6.3)	
	Change	-1.2	(2.6) [a]	0.636
J01M	Post-Policy	16.7	(2.6)	
	Pre-Policy	25.3	(7.8)	
	Change	-8.6	(2.6) [a]	**0.007 [b]**
J01G	Post-Policy	0.2	(0.3)	
	Pre-Policy	0.1	(0.1)	
	Change	+0.1	(0.1) [a]	0.280
J01X	Post-Policy	0.2	(0.1)	
	Pre-Policy	0.1	(0)	
	Change	+0.1	(0) [a]	**0.003 [b]**

Table 2. Effect of COVID-19 Pandemic on Antibiotic Consumption Comparing the Consumption of Q3 2018 till Q1 2020 with the Remining Quarters of 2020 (Q2-Q4, 2020)

Consumption	Period	Mean (SD)		P-value *
Total	Q2-Q4, 2020	191.9	(22.7)	
	Q3, 2018-Q1, 2020	228.7	(30.3)	
	Change	-36.8	(19.7) [a]	0.117
Oral	Q2-Q4, 2020	189.5	(22.5)	
	Q3, 2018-Q1, 2020	226.4	(30.1)	
	Change	-36.8	(19.6) [a]	0.067
Parenteral	Q2-Q4, 2020	3.3	(0.3)	
	Q3, 2018-Q1, 2020	2.9	(0.8)	
	Change	+0.4	(0.5) [a]	0.383
**Consumption by Class**				
J01A	Q2-Q4, 2020	12.9	(6.1)	
	Q3, 2018-Q1, 2020	12.1	(3.9)	
	Change	+0.8	(3.8) [a]	0.833
J01C	Q2-Q4, 2020	111.9	(16.3)	
	Q3, 2018-Q1, 2020	128.7	(14.7)	
	Change	-16.8	(10.4) [a]	0.117
J01D	Q2-Q4, 2020	22.0	(2.4)	
	Q3, 2018-Q1, 2020	39.7	(9.4)	
	Change	-17.7	(5.7) [a]	**0.017** [b]
J01E	Q2-Q4, 2020	2.0	(0.8)	
	Q3, 2018-Q1, 2020	0.6	(0.6)	
	Change	+1.4	(0.4) [a]	**0.033** [b]
J01F	Q2-Q4, 2020	27.5	(2.7)	
	Q3, 2018-Q1, 2020	28.7	(6.1)	
	Change	-1.2	(3.7) [a]	0.667
J01M	Q2-Q4, 2020	14.3	(2.2)	
	Q3, 2018-Q1, 2020	17.8	(2.1)	
	Change	-3.5	(1.5) [a]	0.117
J01G	Q2-Q4, 2020	0.1	(0.02)	
	Q3, 2018-Q1, 2020	0.3	(0.4)	
	Change	-0.2	(0.2) [a]	0.517
J01X	Q2-Q4, 2020	0.2	(0.1)	
	Q3, 2018-Q1, 2020	0.2	(0.1)	
	Change	0	(0.1) [a]	>0.999
